# The Role and Mechanism of HIF‐1α in Regulating A549 Alveolar Epithelial Cell Function Under Hypoxic Conditions

**DOI:** 10.1155/carj/1747792

**Published:** 2026-02-02

**Authors:** Wei Zhao, Kun Wang, Qiuyue Kou, Yeying Feng, Ling Song, Tieyan Wang

**Affiliations:** ^1^ Neonatology Department, The Second Affiliated Hospital of Qiqihar Medical University, Qiqihar, 161000, Heilongjiang, China

**Keywords:** alveolar Type II epithelial cells (ATII cell), angiogenesis, hypoxia, hypoxia-inducible transcription Factor 1 (HIF-1α), vascular endothelial growth factor (VEGF)

## Abstract

**Objective:**

This study aims to investigate the regulatory role and mechanism of hypoxia‐inducible transcription Factor 1 (HIF‐1α) in alveolar epithelial cell function under hypoxic conditions using A549 cells as a surrogate model.

**Methods:**

Human A549 alveolar epithelial cells were used as the experimental model. HIF‐1α expression was modulated by siRNA knockdown or plasmid overexpression. qRT‐PCR quantified HIF‐1α, SP‐C, and vascular endothelial growth factor (VEGF) mRNA levels, and Western blotting evaluated the corresponding proteins and apoptosis‐related markers (cleaved Caspase 3, Bcl‐2, Bax). Cell counting kit 8 (CCK‐8) assessed A549 cell viability, while transwell assays measured migration (uncoated membrane) and invasion (Matrigel‐coated membrane). Terminal deoxynucleotidyl transferase–mediated dUTP nick‐end labeling (TUNEL) staining detected the apoptosis. Enzyme‐linked immunosorbent assay (ELISA) quantified VEGF secretion, and tube‐formation assays evaluated the proangiogenic effects of A549‐conditioned media on human umbilical vein endothelial cells (HUVECs).

**Results:**

Hypoxia markedly increased HIF‐1α and VEGF expression while reducing SP‐C expression in A549 cells. HIF‐1α knockdown decreased VEGF expression and angiogenesis, restored cell viability, and suppressed migration, invasion, and apoptosis. In contrast, HIF‐1α overexpression further enhanced angiogenesis, promoted migration and invasion, and increased apoptosis.

**Conclusions:**

HIF‐1α is a key regulator of hypoxia‐induced functional changes in alveolar epithelial cells, influencing cell viability, migration, invasion, apoptosis, VEGF production, and proangiogenic activity. These findings highlight their potential as a therapeutic target in hypoxia‐related lung injury.

## 1. Introduction

A condition of decreased oxygen availability, hypoxia is a major pathogenic characteristic of many lung disorders, including chronic obstructive pulmonary disease (COPD) and acute respiratory distress syndrome (ARDS) [1, 2]. It frequently results in malfunctioning of alveolar epithelial cells. Therefore, hypoxia has long been considered a key factor affecting the cellular and molecular responses of lung tissues [3]. Alveolar epithelial Type II cells (ATII cells) are essential for surfactant production and alveolar repair, while gas exchange is primarily carried out by Type I epithelial cells, with ATII‐derived surfactant indirectly supporting this process [4–6]. Under hypoxic conditions, cellular responses are primarily regulated by hypoxia‐inducible Factor‐1 alpha (HIF‐1α), a central transcription factor activated under hypoxic conditions that coordinate a range of biological processes, including angiogenesis, cell survival, and metabolism [7–9]. Among the downstream targets of HIF‐1α, vascular endothelial growth factor (VEGF) has emerged as a key mediator of angiogenesis and vascular permeability in hypoxic tissues [10, 11]. In ATII cells, VEGF is not only involved in maintaining alveolar–capillary barrier integrity but also contributes to alveolar repair and regeneration [12, 13].

Studies have shown that hypoxia significantly upregulates the expression of HIF‐1α in ATII cells, thereby increasing VEGF secretion [10, 14]. This pathway is critical for adaptation to hypoxic stress, as VEGF promotes endothelial cell migration and capillary formation, a key process in restoring oxygen supply to damaged lung tissues [15, 16]. However, aberrant activation of the HIF‐1α/VEGF axis is associated with pathological conditions such as pulmonary fibrosis and acute lung injury, where excessive angiogenesis and vascular leakage exacerbate disease progression [17, 18]. In addition to its role in angiogenesis, HIF‐1α affects other cellular processes in ATII cells under hypoxic conditions. For example, it regulates the expression of the surfactant protein SP‐C, which is critical for alveolar stability [19]. Dysregulation of HIF‐1α and its downstream targets, including proapoptotic proteins (e.g., Bax) and antiapoptotic proteins (e.g., Bcl‐2), is associated with increased apoptosis in ATII cells due to prolonged hypoxia [20]. This apoptotic response mediated by the HIF‐1α signaling pathway highlights its dual role in promoting adaptive and pathological processes in hypoxic environments.

Emerging evidence indicates that HIF‐1α‐mediated signaling extends beyond VEGF and apoptosis, affecting ATII cell migration, invasion, and epithelial–mesenchymal transition (EMT). For instance, under hypoxic conditions, HIF‐1α activation is associated with increased epithelial cell invasiveness and migration, contributing to the progression of fibrotic lesions [21]. Despite these findings, the precise mechanisms balancing HIF‐1α‐mediated protective and pathological responses in ATII cells under hypoxia remain poorly understood.

Given the critical role of HIF‐1α in regulating VEGF expression, apoptosis, and cell migration in ATII cells, this study aims to elucidate the molecular mechanisms underlying its function under hypoxic conditions. By systematically investigating these pathways, the research seeks to provide insights into potential therapeutic targets for mitigating lung injury and promoting tissue repair.

## 2. Materials and Methods

### 2.1. Cell Culture and Grouping

The human lung adenocarcinoma epithelial cell line A549 and human umbilical vein endothelial cells (HUVECs) used in this study were obtained from ATCC, and cells at Passages 3–8 (A549) and 3–6 (HUVECs) were used in all experiments to ensure phenotype stability. A549 cells were cultured in high‐glucose DMEM supplemented with 10% fetal bovine serum (FBS) and 1% penicillin–streptomycin (P/S), and maintained under normoxic conditions in an incubator at 37°C with 5% CO_2_. HUVECs were cultured in complete medium (EGM‐2, Lonza) under the same normoxic conditions. A549 cells were allocated into six experimental groups in a schematic manner as follows:

1. Normoxia (21% O_2_);
2. Hypoxia (1% O_2_);
3. Hypoxia + siRNA negative control (siNC);
4. Hypoxia + HIF‐1α knockdown (si‐HIF‐1α);
5. Hypoxia + vector negative control (oeNC);
6. Hypoxia + HIF‐1α overexpression (oe‐HIF‐1α).

HIF‐1α siRNA (si‐HIF‐1α) and its negative control (siNC) were purchased from GenePharma (Shanghai, China). The HIF‐1α overexpression plasmid (oe‐HIF‐1α) and the corresponding vector control (oeNC) were obtained from Vigene Biosciences (Shandong, China). All constructs were sequence‐verified by the manufacturers, and transfections were performed using Lipofectamine 3000 according to the manufacturer’s instructions. Hypoxic conditions were established using a hypoxic incubator (1% O_2_, 5% CO_2_) for a 24‐h exposure period [22]. After the experiments, culture medium and cell samples were collected for subsequent molecular biological assays and functional analyses.

### 2.2. qRT‐PCR

Total RNA from cells in each group was extracted using TRIzol reagent (Invitrogen, USA), and cDNA was synthesized by reverse transcription according to the instructions of PrimeScript RT kit (Takara, Japan). Then, qRT‐PCR amplification was performed according to the specific operation of SYBR Green detection kit (Takara, Japan). The amplification program was 95°C for 30 s, 95°C for 5 s, 60°C for 30 s, and 40 cycles. Primers were purchased from Shanghai Sangon Bioengineering Technology Services Co., Ltd. (Table 1). Using β‐actin as the internal reference gene, the 2^−ΔΔCt^ method was used to calculate the relative expression of the target gene, and the relative expression levels of the mRNA of each group of genes were displayed in a histogram. Three biological replicates were performed for each reaction.

**TABLE 1:** qPCR primers used in this study.

Name	Forward primer (5′‐3′)	Reverse primer (5′‐3′)
HIF‐1α	CAG​TCG​ACA​CAG​CCT​GGA​TA	CCA​CCT​CTT​TTG​GCA​AGC​AT
VEGF	GCT​ACT​GCC​ATC​CAA​TCG​AG	CTT​GGT​GAG​GTT​TGA​TCC​GC
SP‐C	GCA​GCA​AAG​AGG​TCC​TGA​TG	CAT​CTC​CGT​GTG​TTT​CTG​GC
β‐actin	CCC​TGG​AGA​AGA​GCT​ACG​AG	CGT​ACA​GGT​CTT​TGC​GGA​TG

### 2.3. CCK‐8

CCK‐8 kit (Beyotime Company, China) was used to detect the viability of A549 cells in each group. Each experimental group was seeded in a 96‐well plate, and the number of cells seeded in each well was 2 × 10^3^. After the cells adhered, they were processed according to the experimental design conditions. At 0, 24, 48, and 72, add 10 μL of CCK‐8 reagent to each well and incubate in the dark for 2 h. The absorbance (OD value) of each well was then read using a microplate reader (Thermo Fisher Scientific, USA) at a wavelength of 450 nm. The cell viability level was evaluated by comparing the changes in OD values of each group.

### 2.4. Transwell Assay

The transwell assay was used to evaluate the migration and invasion abilities of A549 cells in each group. For the migration assay, a cell suspension (2 × 10^4^ cells/well) was seeded into the upper chamber of transwell inserts without Matrigel coating, using serum‐free medium containing 0.1% FBS. The lower chamber was filled with medium containing 10% FBS as a chemoattractant. For the invasion assay, Matrigel was precoated on the bottom of the upper chamber, and cells were seeded in the same manner. All setups were incubated at 37°C with 5% CO_2_ for 24 h. After incubation, nonmigrated or noninvaded cells in the upper chamber were gently removed with a cotton swab, while cells that had migrated or invaded through the membrane were fixed and stained with 0.1% crystal violet. The stained transwell membranes were observed and imaged using an Olympus IX73 inverted microscope (Olympus, Japan), and the number of cells in five randomly selected fields was counted.

### 2.5. TUNEL Assay

TUNEL kit (Beyotime, China) was used to evaluate the apoptosis level of A549 cells under different experimental conditions. First, the treated A549 cells were gently washed twice with PBS and fixed with 4% paraformaldehyde for 15 min. Subsequently, 0.2% Triton X‐100 was added to permeabilize the cell membrane and was incubated at room temperature for 5 min. After fixation and permeabilization are completed, follow the instructions of the TUNEL detection kit, add TUNEL reaction solution to the cells, and incubate the cells at 37°C in the dark for 1 h. After incubation, the cells were gently washed with PBS, and the fluorescent signals were captured using an Olympus IX73 fluorescence microscope (Olympus, Japan). The green fluorescence signal represents apoptotic cells. Five fields of view were randomly selected in each group to count the number of fluorescence‐positive cells, and the apoptosis rate (the number of apoptotic cells as a percentage of the total number of cells) was calculated.

### 2.6. Western Blot Analysis

Western blot was performed to analyze the expression of target proteins in A549 cells from different groups. Cells were washed twice with cold PBS, lysed with RIPA buffer containing 1% phenylmethylsulfonyl fluoride (PMSF), and incubated on ice for 30 min. The lysates were centrifuged, and the supernatants were collected as total protein extracts. Protein concentrations were determined using a bicinchoninic acid (BCA) protein assay kit. Equal amounts of protein (30 μg/lane) were separated on 10% SDS‐PAGE gels and transferred onto polyvinylidene difluoride (PVDF) membranes (Millipore, USA). After blocking with 5% nonfat milk at room temperature for 2 h, the membranes were incubated overnight at 4°C with primary antibodies diluted in tris‐buffered saline containing 0.1% Tween‐20 (TBST). The primary antibodies used in this study were HIF‐1α (ab179483, 1:1000), SP‐C (ab90716, 1:1000), VEGF‐A (ab46154, 1:1000), Bcl‐2 (ab182858, 1:1000), Bax (ab182733, 1:1000), cleaved Caspase 3 (ab32042, 1:1000), and β‐actin (ab8226, 1:1000), all purchased from Abcam (UK).

The next day, the membranes were washed three times with TBST and incubated with an HRP‐conjugated goat anti‐rabbit IgG H&L secondary antibody (ab205718, Abcam; 1:5000) at room temperature for 1 h. After washing, protein bands were visualized using an enhanced chemiluminescence (ECL) detection system, and band intensities were quantified using ImageJ software (ImageJ v1.53).

### 2.7. ELISA Detection

The concentration of VEGF‐A in the culture medium of A549 cells was measured using a human VEGF ELISA kit (PV963, Beyotime) according to the manufacturer’s instructions. A standard curve was generated using seven concentrations (0, 62.5, 125, 250, 500, 1000, and 2000  pg/mL), and absorbance was measured at 450 nm. VEGF‐A levels were calculated based on the standard curve. The minimum detectable concentration of the assay was 29.4 pg/mL. After treating the cells according to experimental groups, the culture medium was collected and centrifuged at 4°C for 15 min at 3000 rpm to obtain the supernatant. The ELISA assay was then performed according to the manufacturer’s instructions. Standard samples and supernatants were added to the 96‐well plates precoated with VEGF antibodies and incubated at 37°C for 2 h. After incubation, the wells were washed three times with washing buffer, followed by the addition of the enzyme conjugate. The plate was incubated at 37°C for 30 min. After another washing step, substrate solution was added, and the plate was incubated in the dark at 37°C for 15 min. Finally, a stop solution was added to terminate the reaction, and the optical density (OD) of each well was measured at 450 nm using a microplate reader. VEGF concentrations in the samples were calculated based on a standard curve.

### 2.8. Tube Formation Assay

The tube formation assay was used to evaluate the effect of each group of culture medium on the angiogenic ability of HUVECs. First, precooled Matrigel was evenly spread at the bottom of a 96‐well plate (50 μL/well) and incubated at 37°C for 30 min to solidify. Then, a suspension of HUVECs (2 × 10^4^ cells/well) was mixed with the conditioned medium from each A549 cell group and added to the Matrigel‐coated wells. For the positive control group, complete medium containing 50 ng/mL VEGF was used [23], while serum‐free medium without VEGF served as the negative control. The cells were incubated at 37°C with 5% CO_2_ for 6–8 h. Tube‐like structures were photographed using an Olympus IX73 inverted microscope (Olympus, Japan). The total tube length, number of branch points, and network distribution were quantified using ImageJ software to evaluate the tube formation ability of HUVECs.

### 2.9. Statistical Analysis

Experimental data were analyzed using GraphPad statistical software (GraphPad Prism 8.0). Student’s *t*‐test was used for comparisons between two groups, and one‐way analysis of variance (ANOVA) followed by Tukey’s post hoc test was applied for comparisons among multiple groups. Data are presented as the mean ± standard deviation (SD) from at least three independent experiments, with *p* < 0.05 considered statistically significant.

## 3. Results

### 3.1. Expression of HIF‐1α, SP‐C, and VEGF in A549 Cells in Different Treatment Groups

Using qRT‐PCR and Western blot, we examined the expression levels of HIF‐1α, SP‐C, and VEGF in A549 cells under different treatment conditions to investigate the regulatory role of HIF‐1α in ATII cell function under hypoxic conditions. Hypoxia markedly increased the mRNA and protein expression of HIF‐1α and VEGF compared with the normoxia group (*p* < 0.01, Figure 1), whereas SP‐C expression was significantly decreased (*p* < 0.01). HIF‐1α knockdown reduced HIF‐1α and VEGF levels (*p* < 0.01) and restored SP‐C expression (*p* < 0.01) relative to the hypoxia group. In contrast, HIF‐1α overexpression further elevated HIF‐1α and VEGF expression and further suppressed SP‐C (*p* < 0.01). No significant differences were observed among the hypoxia, siNC, and oeNC groups. These findings demonstrate that hypoxia upregulates HIF‐1α and VEGF while downregulating SP‐C and that modulating HIF‐1α expression directly influences VEGF and SP‐C levels under hypoxic conditions.

FIGURE 1The mRNA and protein expression levels of HIF‐1α, SP‐C, and VEGF in A549 cells in different treatment groups. (a–c) qRT‐PCR analysis of HIF‐1α, SP‐C, and VEGF expression. (d) Western blot analysis of protein levels. Data are presented as mean ± SD from three independent biological replicates (*n* = 3). Statistical analysis was performed using one‐way ANOVA with Tukey’s post hoc test. ^∗∗^ *p* < 0.01 vs. N group; ^##^ *p* < 0.01 vs. H group.

**Figure:**
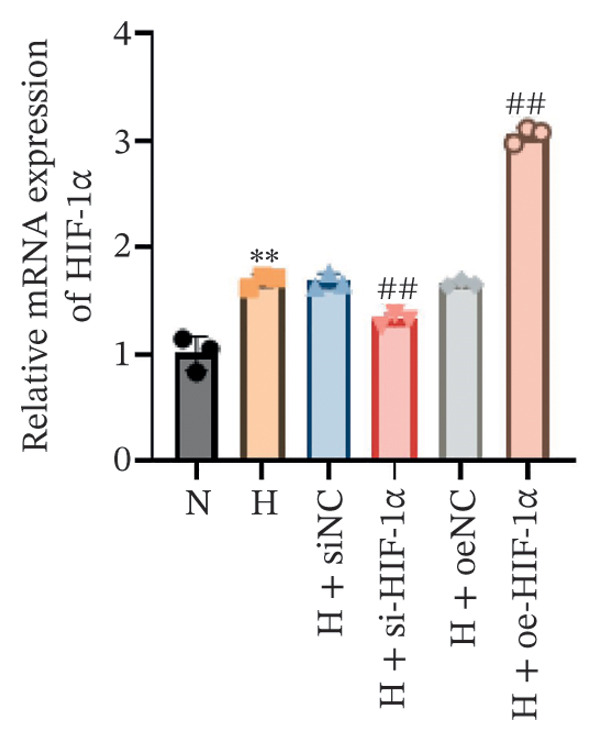
(a)

**Figure:**
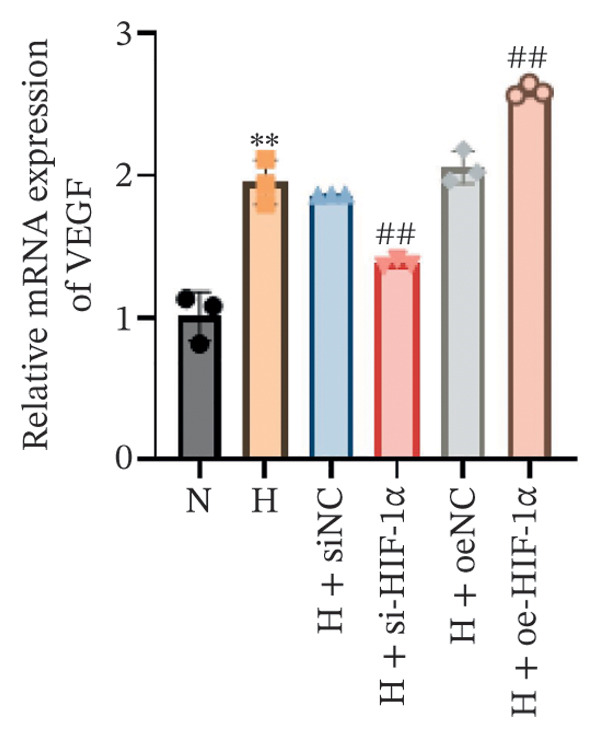
(b)

**Figure:**
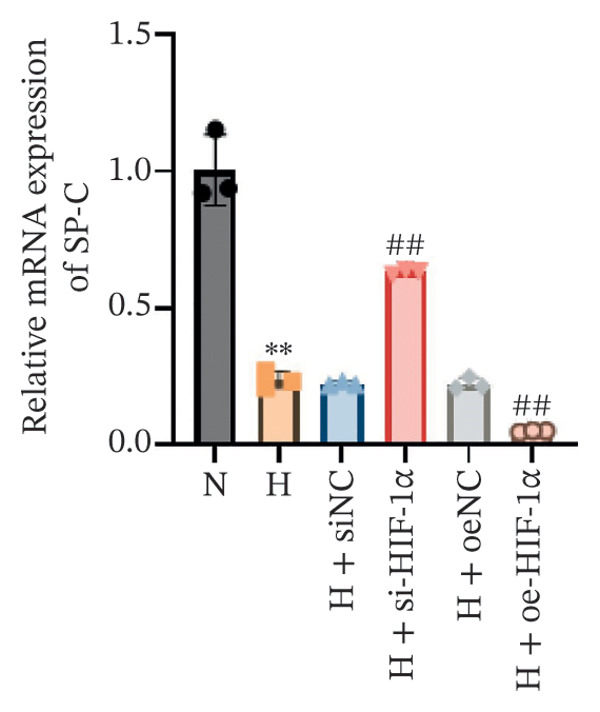
(c)

**Figure:**
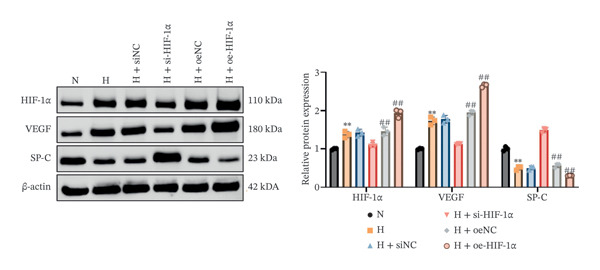
(d)

### 3.2. The Regulatory Effect of HIF‐1α on the Viability, Migration, and Invasion of A549 Cells

In order to study the effect of HIF‐1α on the viability, migration, and invasion ability of A549 cells under hypoxic conditions, we performed CCK‐8 detection and transwell experiments. Cell viability was significantly reduced under hypoxia compared with the normoxia group (*p* < 0.01, Figure 2), whereas migration and invasion were markedly enhanced (*p* < 0.01). HIF‐1α knockdown restored cell viability and suppressed migration and invasion relative to the hypoxia group (all *p* < 0.01). In contrast, HIF‐1α overexpression further decreased viability and promoted both migration and invasion (*p* < 0.01). No significant differences were observed among the hypoxia, siNC, and oeNC groups. These findings indicate that HIF‐1α plays a crucial role in regulating A549 cell viability, migration, and invasion under hypoxic conditions.

FIGURE 2Effect of HIF‐1α on the viability, migration, and invasion of A549 cells. (a) CCK‐8 assay of cell viability. (b) Transwell migration assay. (c) Transwell invasion assay. Data represent mean ± SD from three biological replicates, with three technical replicates for CCK‐8. Statistical analysis: one‐way ANOVA with Tukey’s post hoc test. ^∗∗^ *p* < 0.01 vs. N group; ^##^ *p* < 0.01 vs. H group.

**Figure:**
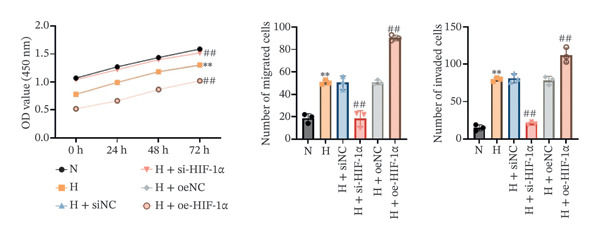
(a)

**Figure:**
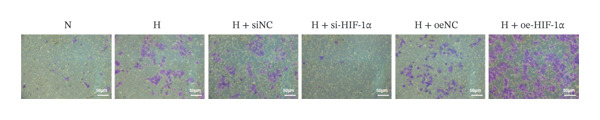
(b)

**Figure:**
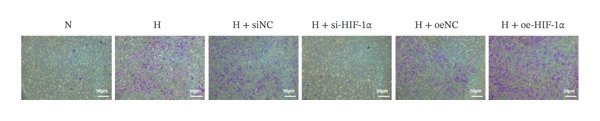
(c)

### 3.3. Effect of HIF‐1α on A549 Cell Apoptosis Under Hypoxic Conditions

Related experiments (TUNEL detection and Western blot analysis) evaluated the regulatory effect of HIF‐1α on A549 cell apoptosis under hypoxic conditions. Apoptosis was markedly increased in hypoxic A549 cells compared with the normoxia group (*p* < 0.01, Figure 3), accompanied by upregulation of cleaved Caspase 3 and Bax and downregulation of Bcl‐2 (all *p* < 0.01). HIF‐1α knockdown significantly reduced apoptosis (*p* < 0.01), decreased cleaved Caspase 3 and Bax levels (*p* < 0.01), and increased Bcl‐2 expression (*p* < 0.01). In contrast, HIF‐1α overexpression further elevated apoptosis (*p* < 0.01), with higher cleaved Caspase 3 and Bax levels and lower Bcl‐2 expression than the hypoxia group (all *p* < 0.01). No significant differences were observed between the hypoxia, siNC, and oeNC groups. These findings demonstrate that HIF‐1α regulates apoptosis in A549 cells under hypoxic conditions by modulating apoptosis‐related proteins.

FIGURE 3Effects of different treatments on apoptosis and apoptosis‐related proteins in A549 cells. (a) TUNEL staining of apoptosis (scale bar: 50 μm). (b) Western blot analysis of cleaved Caspase 3, Bax, and Bcl‐2. Data are shown as mean ± SD from three biological replicates (*n* = 3). Statistical analysis: one‐way ANOVA followed by Tukey’s post hoc test. ^∗∗^ *p* < 0.01 vs. N group; ^##^ *p* < 0.01 vs. H group.

**Figure:**
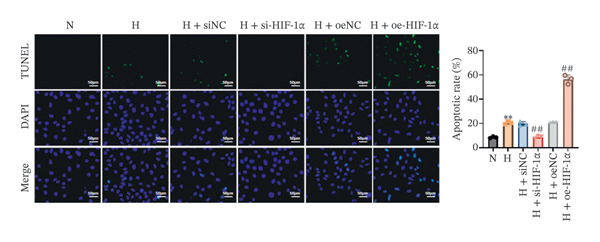
(a)

**Figure:**
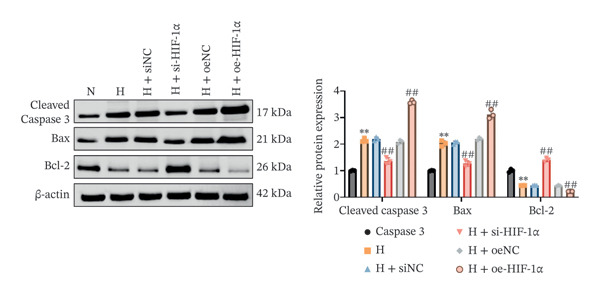
(b)

### 3.4. Regulation of VEGF Secretion and Angiogenesis by HIF‐1α

This study aimed to evaluate the regulatory role of HIF‐1α on VEGF secretion and angiogenesis under hypoxic conditions. Hypoxia significantly increased VEGF secretion (*p* < 0.01, Figure 4(b)), while HIF‐1α knockdown markedly reduced VEGF release (*p* < 0.01) and HIF‐1α overexpression further enhanced it (*p* < 0.01). Consistently, hypoxia markedly promoted angiogenesis, as indicated by increased branch points and total tube length (*p* < 0.01, Figure 4(a)). HIF‐1α knockdown attenuated this proangiogenic effect (*p* < 0.01), whereas HIF‐1α overexpression further amplified angiogenesis, with both branch points and total tube length significantly exceeding those of the hypoxia group (*p* < 0.01).

FIGURE 4Effect of HIF‐1α on VEGF secretion and angiogenesis under hypoxic conditions. (a) Tube‐formation assay of HUVEC angiogenesis (scale bar: 100 μm). (b) ELISA quantification of VEGF secretion. Data represent mean ± SD from three independent experiments, each with three technical replicates. Statistical analysis: one‐way ANOVA with Tukey’s post hoc test. ^∗∗^ *p* < 0.01 vs. N group; ^##^ *p* < 0.01 vs. H group.

**Figure:**
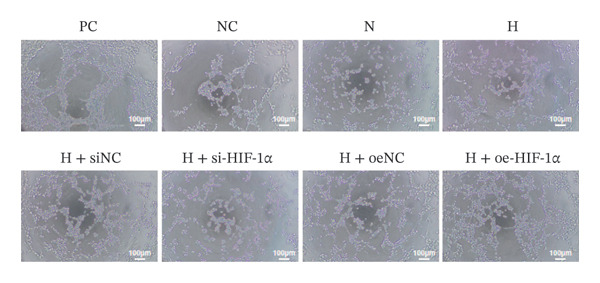
(a)

**Figure:**
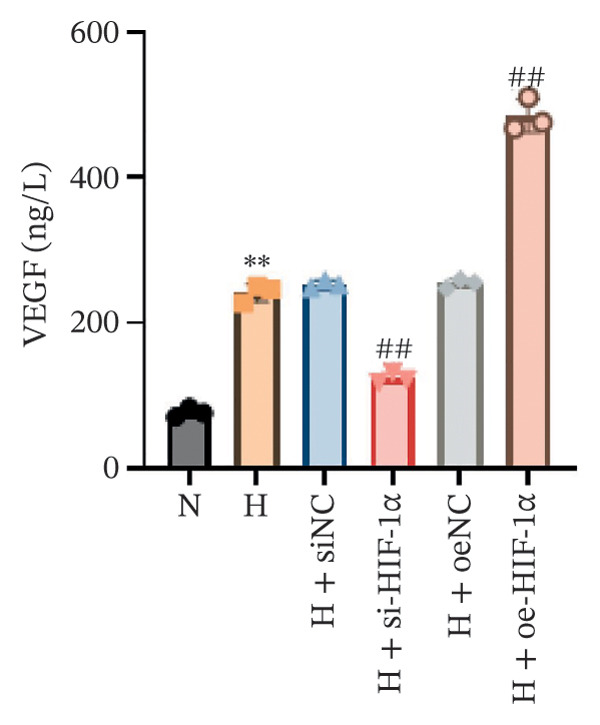
(b)

## 4. Discussion

This study provides comprehensive insights into the regulatory role of HIF‐1α in ATII cells represented by the A549 cell line under hypoxic conditions. Our results show that hypoxia significantly induces the expression of HIF‐1α, leading to changes in cell function, including changes in VEGF secretion, cell viability, migration, invasion, apoptosis, and angiogenic capacity. These findings elucidate the dual role of HIF‐1α as a protective and pathological mediator in the hypoxic lung environment.

An important observation of this study is that HIF‐1α induces an increase in VEGF expression under hypoxic conditions, which emphasizes its role in promoting angiogenesis. VEGF promotes endothelial migration and capillary formation, processes necessary for restoring oxygen supply to damaged lung tissue [24]. However, as observed in our HIF‐1α overexpression model, excess VEGF production exacerbates vascular permeability and may lead to pathological angiogenesis. These findings are consistent with previous studies linking VEGF overexpression to edema and the progression of diseases such as pulmonary fibrosis and ARDS [25–27]. The balance between beneficial and detrimental VEGF signaling remains critical for designing therapeutic strategies targeting HIF‐1α. Second, this study also highlights the profound impact of HIF‐1α on the regulation of apoptosis in ATII cells. Hypoxia‐induced activation of HIF‐1α increases apoptotic markers, including cleaved Caspase 3 and Bax while reducing antiapoptotic Bcl‐2 levels. These findings suggest that HIF‐1α‐driven apoptosis has a dual role: clearing damaged cells while potentially impairing tissue repair during sustained hypoxia. This observation correlates with earlier reports that prolonged HIF‐1α activity promotes ATII apoptosis, leading to disruption of alveolar function and disease progression [28, 29].

In addition to its role in apoptosis, our study reveals that HIF‐1α also influences cell migration and invasion, which are critical processes in tissue remodeling. Hypoxia‐induced HIF‐1α activation enhances these invasive properties, associated with an increase in EMT. EMT is not restricted to fibrotic lung diseases; it is also widely observed in chronic airway disorders such as asthma and COPD, where it reflects ongoing airway remodeling and contributes to pathological progression [30, 31]. In our model, hypoxia‐induced HIF‐1α activation may enhance EMT‐related migratory and invasive phenotypes, consistent with its broader role in lung remodeling. Furthermore, related research suggests that HIF‐1α‐mediated EMT is amplified through interactions with inflammatory cytokines and ROS, highlighting the intricate interplay between hypoxia and inflammation in lung remodeling [32, 33]. Notably, our study identified a novel role of HIF‐1α in regulating SP‐C expression. SP‐C is essential for maintaining alveolar stability and surfactant homeostasis [34]. Hypoxia‐induced HIF‐1α suppresses SP‐C expression, potentially impairing alveolar integrity and gas exchange efficiency [35]. This observation aligns with recent studies linking SP‐C downregulation to alveolar collapse and impaired lung function in hypoxic environments. The therapeutic implications of these findings are substantial. In our study, HIF‐1α inhibition reduced VEGF levels, restored SP‐C expression, and mitigated apoptosis, migration, and invasion under hypoxic conditions. This suggests that targeting HIF‐1α could be a promising strategy to balance its protective and pathological roles. However, excessive inhibition of HIF‐1α may impede its beneficial effects, such as angiogenesis and tissue repair [36]. Therefore, fine‐tuning HIF‐1α activity is crucial for developing effective therapies for hypoxia‐related lung diseases.

Despite these advancements, our study has limitations. The A549 cell line, while commonly used as an ATII cell model, does not fully replicate the physiology of primary ATII cells [37]. Additionally, the interactions of HIF‐1α with other signaling pathways, such as NF‐κB and TGF‐β, ROS, and inflammatory cytokines under hypoxic conditions, warrant further investigation [38, 39]. Future studies should focus on in vivo models to validate these findings and explore the therapeutic potential of HIF‐1α modulators in hypoxia‐induced lung injury.

In conclusion, this study expands our understanding of the multifaceted roles of HIF‐1α in regulating ATII cell responses under hypoxic conditions. By elucidating its effects on VEGF secretion, apoptosis, migration, invasion, and SP‐C expression, we underscore HIF‐1α as a central mediator of protective and pathological responses in the lung. These insights lay the groundwork for targeted interventions to alleviate hypoxic lung injury and promote repair.

## 5. Conclusion

In conclusion, HIF‐1α regulates key hypoxia‐induced changes in A549 cells, including alterations in VEGF secretion, apoptosis, migration, invasion, and SP‐C expression. These findings identify HIF‐1α as an important mediator of cellular responses to hypoxia, while further studies are required to determine their relevance in vivo.

**Nomenclature**
HIF‐1α: Hypoxia‐inducible transcription Factor 1
ATII cell: Alveolar Type II epithelial cell
VEGF: Vascular endothelial growth factor
COPD: Chronic obstructive pulmonary disease
ARDS: Acute respiratory distress syndrome
EMT: Epithelial–mesenchymal transition
HUVECs: Human umbilical vein endothelial cells
FBS: Fetal bovine serum
P/S: Penicillin–streptomycin

## Author Contributions

All authors contributed to the study conception and design. Material preparation, data collection, and analysis were performed by Qiuyue Kou, Yeying Feng, and Ling Song. The first draft of this manuscript was written by Wei Zhao, Kun Wang, and Tieyan Wang, and all authors commented on previous versions of the manuscript.

## Funding

This study was supported by Qiqihar Science and Technology Plan Project (no. LSFGG‐2023074).

## Disclosure

All authors read and approved the final manuscript.

## Ethics Statement

The authors have nothing to report.

## Consent

The authors have nothing to report.

## Conflicts of Interest

The authors declare no conflicts of interest.

## Data Availability

The datasets used and/or analyzed during the current study are available from the corresponding author on reasonable request.

## References

[1] Grek C. L. , Newton D. A. , Spyropoulos D. D. , and Baatz J. E. , Hypoxia Up-Regulates Expression of Hemoglobin in Alveolar Epithelial Cells, American Journal of Respiratory Cell and Molecular Biology. (2011) 44, no. 4, 439–447, 10.1165/rcmb.2009-0307oc, 2-s2.0-79953732001. 20508070 PMC3095918

[2] Stenmark K. R. , Fagan K. A. , and Frid M. G. , Hypoxia-Induced Pulmonary Vascular Remodeling: Cellular and Molecular Mechanisms, Circulation Research. (2006) 99, no. 7, 675–691, 10.1161/01.res.0000243584.45145.3f, 2-s2.0-33749345684. 17008597

[3] Sturrock A. , Woller D. , Freeman A. , Sanders K. , and Paine R.3rd, Consequences of Hypoxia for the Pulmonary Alveolar Epithelial Cell Innate Immune Response, Journal of Immunology. (2018) 201, no. 11, 3411–3420, 10.4049/jimmunol.1701387, 2-s2.0-85056734230. PMC6246786 30381478

[4] Mason R. J. , Biology of Alveolar Type II Cells, Respirology. (2006) 11, no. Suppl, S12–S15, 10.1111/j.1440-1843.2006.00800.x, 2-s2.0-33645106888. 16423262

[5] Ruaro B. , Salton F. , Braga L. et al., The History and Mystery of Alveolar Epithelial Type II Cells: Focus on Their Physiologic and Pathologic Role in Lung, International Journal of Molecular Sciences. (2021) 22, no. 5, 10.3390/ijms22052566. PMC7961977 33806395

[6] Whitsett J. A. , Wert S. E. , and Weaver T. E. , Alveolar Surfactant Homeostasis and the Pathogenesis of Pulmonary Disease, Annual Review of Medicine. (2010) 61, no. 1, 105–119, 10.1146/annurev.med.60.041807.123500, 2-s2.0-77951909085. PMC4127631 19824815

[7] Porel P. , Bala K. , and Aran K. R. , Exploring the Role of HIF-1α on Pathogenesis in Alzheimer’s Disease and Potential Therapeutic Approaches, Inflammopharmacology. (2024) . 10.1007/s10787-024-01585-x 39465478

[8] Ubaid S. , Kashif M. , Laiq Y. , Nayak A. K. , Kumar V. , and Singh V. , Targeting HIF-1α in Sickle Cell Disease and Cancer: Unraveling Therapeutic Opportunities and Risks, Expert Opinion on Therapeutic Targets. (2024) 28, no. 5, 357–373, 10.1080/14728222.2024.2367640. 38861226

[9] Monaci S. , Coppola F. , Filippi I. , Falsini A. , Carraro F. , and Naldini A. , Targeting Hypoxia Signaling Pathways in Angiogenesis, Frontiers in Physiology. (2024) 15, 10.3389/fphys.2024.1408750. PMC11079266 38725568

[10] Pham I. , Uchida T. , Planes C. et al., Hypoxia Upregulates VEGF Expression in Alveolar Epithelial Cells In Vitro and In Vivo, American Journal of Physiology-Lung Cellular and Molecular Physiology. (2002) 283, no. 5, L1133–L1142, 10.1152/ajplung.00464.2001. 12376368

[11] Chen Y. , Di M. , Tang Y. et al., Epstein-Barr Virus Causes Vascular Abnormalities in Epithelial Malignancies Through Upregulating ANXA3-HIF-1α-VEGF Pathway, Oncogene. (2024) 43, no. 28, 2143–2159, 10.1038/s41388-024-03061-w. 38778160

[12] McClendon J. , Jansing N. L. , Redente E. F. et al., Hypoxia-Inducible Factor 1α Signaling Promotes Repair of the Alveolar Epithelium After Acute Lung Injury, The American Journal of Pathology. (2017) 187, no. 8, 1772–1786, 10.1016/j.ajpath.2017.04.012, 2-s2.0-85023780988. 28618253 PMC5530913

[13] Mammoto A. and Mammoto T. , Vascular Niche in Lung Alveolar Development, Homeostasis, and Regeneration, Frontiers in Bioengineering and Biotechnology. (2019) 7, 10.3389/fbioe.2019.00318. PMC6861452 31781555

[14] Tuder R. M. , Flook B. E. , and Voelkel N. F. , Increased Gene Expression for VEGF and the VEGF Receptors KDR/Flk and Flt in Lungs Exposed to Acute or to Chronic Hypoxia. Modulation of Gene Expression by Nitric Oxide, The Journal of Clinical Investigation. (1995) 95, no. 4, 1798–1807, 10.1172/jci117858, 2-s2.0-0028940664. 7706486 PMC295709

[15] Lokmic Z. , Musyoka J. , Hewitson T. D. , and Darby I. A. , Jeon K. W. , Chapter Three-Hypoxia and Hypoxia Signaling in Tissue Repair and Fibrosis, International Review of Cell and Molecular Biology, 2012, 296, Academic Press, 139–185, 10.1016/B978-0-12-394307-1.00003-5, 2-s2.0-84860424872. 22559939

[16] Yamaji-Kegan K. , Su Q. , Angelini D. J. , Champion H. C. , and Johns R. A. , Hypoxia-Induced Mitogenic Factor Has Proangiogenic and Proinflammatory Effects in the Lung via VEGF and VEGF receptor-2, American Journal of Physiology-Lung Cellular and Molecular Physiology. (2006) 291, no. 6, L1159–L1168, 10.1152/ajplung.00168.2006, 2-s2.0-33845443200. 16891392

[17] Yang F. , He Z. , Chu Z. et al., An Active Peptide From Yak Inhibits Hypoxia-Induced Lung Injury via Suppressing VEGF/MAPK/inflammatory Signaling, Redox Biology. (2024) 75, 10.1016/j.redox.2024.103252. PMC11255109 38925040

[18] Zhou Y. , Zhu X. , Cui H. et al., The Role of the VEGF Family in Coronary Heart Disease, Frontiers in Cardiovascular Medicine. (2021) 8, 10.3389/fcvm.2021.738325. PMC8421775 34504884

[19] Ito Y. , Ahmad A. , Kewley E. , and Mason R. J. , Hypoxia-Inducible Factor Regulates Expression of Surfactant Protein in Alveolar Type II Cells In Vitro, American Journal of Respiratory Cell and Molecular Biology. (2011) 45, no. 5, 938–945, 10.1165/rcmb.2011-0052oc, 2-s2.0-80555143054. 21454802 PMC3262688

[20] Wang B. , He J. , Cui Y. et al., The HIF-1α/EGF/EGFR Signaling Pathway Facilitates the Proliferation of Yak Alveolar Type II Epithelial Cells in Hypoxic Conditions, International Journal of Molecular Sciences. (2024) 25, no. 3, 10.3390/ijms25031442. PMC10855765 38338723

[21] Kim J. , Kim B. , Kim S. M. et al., Hypoxia-Induced Epithelial-to-Mesenchymal Transition Mediates Fibroblast Abnormalities via ERK Activation in Cutaneous Wound Healing, International Journal of Molecular Sciences. (2019) 20, no. 10, 10.3390/ijms20102546, 2-s2.0-85067290070. PMC6566997 31137604

[22] Bai J. , Xiao X. , Zhang X. et al., Erythropoietin Inhibits Hypoxia-Induced epithelial-to-mesenchymal Transition via Upregulation of miR-200b in HK-2 Cells, Cellular Physiology and Biochemistry. (2017) 42, no. 1, 269–280, 10.1159/000477327, 2-s2.0-85020186738. 28535509

[23] Ferla R. , Bonomi M. , Otvos L.Jr., and Surmacz E. , Glioblastoma-Derived Leptin Induces Tube Formation and Growth of Endothelial Cells: Comparison with VEGF Effects, BMC Cancer. (2011) 11, no. 1, 10.1186/1471-2407-11-303, 2-s2.0-79960561552. PMC3146945 21771332

[24] Eldridge L. and Wagner E. M. , Angiogenesis in the Lung, The Journal of physiology. (2019) 597, no. 4, 1023–1032, 10.1113/jp275860, 2-s2.0-85053196883. 30022479 PMC6376070

[25] Mura M. , dos Santos C. C. , Stewart D. , and Liu M. , Vascular Endothelial Growth Factor and Related Molecules in Acute Lung Injury, Journal of Applied Physiology. (2004) 97, no. 5, 1605–1617, 10.1152/japplphysiol.00202.2004, 2-s2.0-7044233292. 15475552

[26] Barratt S. L. , Flower V. A. , Pauling J. D. , and Millar A. B. , VEGF (Vascular Endothelial Growth Factor) and Fibrotic Lung Disease, International Journal of Molecular Sciences. (2018) 19, no. 5, 10.3390/ijms19051269, 2-s2.0-85046080862. PMC5983653 29695053

[27] Herrero R. , Sanchez G. , and Lorente J. A. , New Insights Into the Mechanisms of Pulmonary Edema in Acute Lung Injury, Annals of Translational Medicine. (2018) 6, no. 2, 10.21037/atm.2017.12.18. PMC5799138 29430449

[28] Krick S. , Eul B. G. , Hänze J. et al., Role of Hypoxia-Inducible Factor-Lalpha in Hypoxia-Induced Apoptosis of Primary Alveolar Epithelial Type II Cells, American Journal of Respiratory Cell and Molecular Biology. (2005) 32, no. 5, 395–403, 10.1165/rcmb.2004-0314OC, 2-s2.0-18244366585. 15695738

[29] Delbrel E. , Soumare A. , Naguez A. et al., HIF-1α Triggers ER Stress and CHOP-Mediated Apoptosis in Alveolar Epithelial Cells, a Key Event in Pulmonary Fibrosis, Scientific Reports. (2018) 8, no. 1, 10.1038/s41598-018-36063-2, 2-s2.0-85058731303. PMC6299072 30560874

[30] Brasier A. R. , Zhao Y. , and Chung K. F. , Editorial: Mucosal Adaptations to Chronic Airway Injury: Mechanisms and Interrelationships of Epithelial Plasticity on Innate Immunity and Airway Remodeling, Frontiers in Immunology. (2024) 15, 10.3389/fimmu.2024.1435120. PMC11263199 39044815

[31] Li S. , Liu Z. , Jiao X. et al., Selpercatinib Attenuates Bleomycin-Induced Pulmonary Fibrosis by Inhibiting the TGF-β1 Signaling Pathway, Biochemical Pharmacology. (2024) 225, 10.1016/j.bcp.2024.116282. 38762147

[32] Zhou G. , Dada L. A. , Wu M. et al., Hypoxia-Induced Alveolar Epithelial-Mesenchymal Transition Requires Mitochondrial ROS and Hypoxia-Inducible Factor 1, American Journal of Physiology-Lung Cellular and Molecular Physiology. (2009) 297, no. 6, L1120–L1130, 10.1152/ajplung.00007.2009, 2-s2.0-71949128382. 19801454 PMC2793183

[33] Liu M. , Ning X. , Li R. et al., Signalling Pathways Involved in Hypoxia-Induced Renal Fibrosis, Journal of Cellular and Molecular Medicine. (2017) 21, no. 7, 1248–1259, 10.1111/jcmm.13060, 2-s2.0-85010747967. 28097825 PMC5487923

[34] Sehlmeyer K. , Ruwisch J. , Roldan N. , and Lopez-Rodriguez E. , Alveolar Dynamics and Beyond-The Importance of Surfactant Protein C and Cholesterol in Lung Homeostasis and Fibrosis, Frontiers in Physiology. (2020) 11, 10.3389/fphys.2020.00386. PMC7213507 32431623

[35] Suresh M. V. , Ramakrishnan S. K. , Thomas B. et al., Activation of Hypoxia-Inducible Factor-1α in Type 2 Alveolar Epithelial Cell is a Major Driver of Acute Inflammation Following Lung Contusion, Critical Care Medicine. (2014) 42, no. 10, e642–e653, 10.1097/ccm.0000000000000488, 2-s2.0-84914176676. 25014067 PMC4245055

[36] Carmeliet P. , Dor Y. , Herbert J.-M. et al., Role of HIF-1α in Hypoxia-Mediated Apoptosis, Cell Proliferation and Tumour Angiogenesis, Nature. (1998) 394, no. 6692, 485–490, 10.1038/28867, 2-s2.0-0032581277. 9697772

[37] Sporty J. L. , Horálková L. , and Ehrhardt C. , In Vitro Cell Culture Models for the Assessment of Pulmonary Drug Disposition, Expert Opinion on Drug Metabolism and Toxicology. (2008) 4, no. 4, 333–345, 10.1517/17425255.4.4.333, 2-s2.0-44349119818. 18433340

[38] Korbecki J. , Simińska D. , Gąssowska-Dobrowolska M. et al., Chronic and Cycling Hypoxia: Drivers of Cancer Chronic Inflammation Through HIF-1 and NF-κB Activation: A Review of the Molecular Mechanisms, International Journal of Molecular Sciences. (2021) 22, no. 19. 10.3390/ijms221910701 PMC8509318 34639040

[39] Olson N. and van der Vliet A. , Interactions Between Nitric Oxide and Hypoxia-Inducible Factor Signaling Pathways in Inflammatory Disease, Nitric Oxide: Biology and Chemistry. (2011) 25, no. 2, 125–137, 10.1016/j.niox.2010.12.010, 2-s2.0-80051549232. 21199675 PMC3090692

